# Supplementary material for: Evidence supported by Mendelian randomization: impact on inflammatory factors in knee osteoarthritis
Source: Front Med (Lausanne). 2024 May 28;11:1382836. doi: 10.3389/fmed.2024.1382836 (PMC11165061; doi:10.3389/fmed.2024.1382836)
Supplement: Supplementary file 1 [file Data_Sheet_1.ZIP › data/GCST90274794_5e6/794.scatter_plot.pdf]

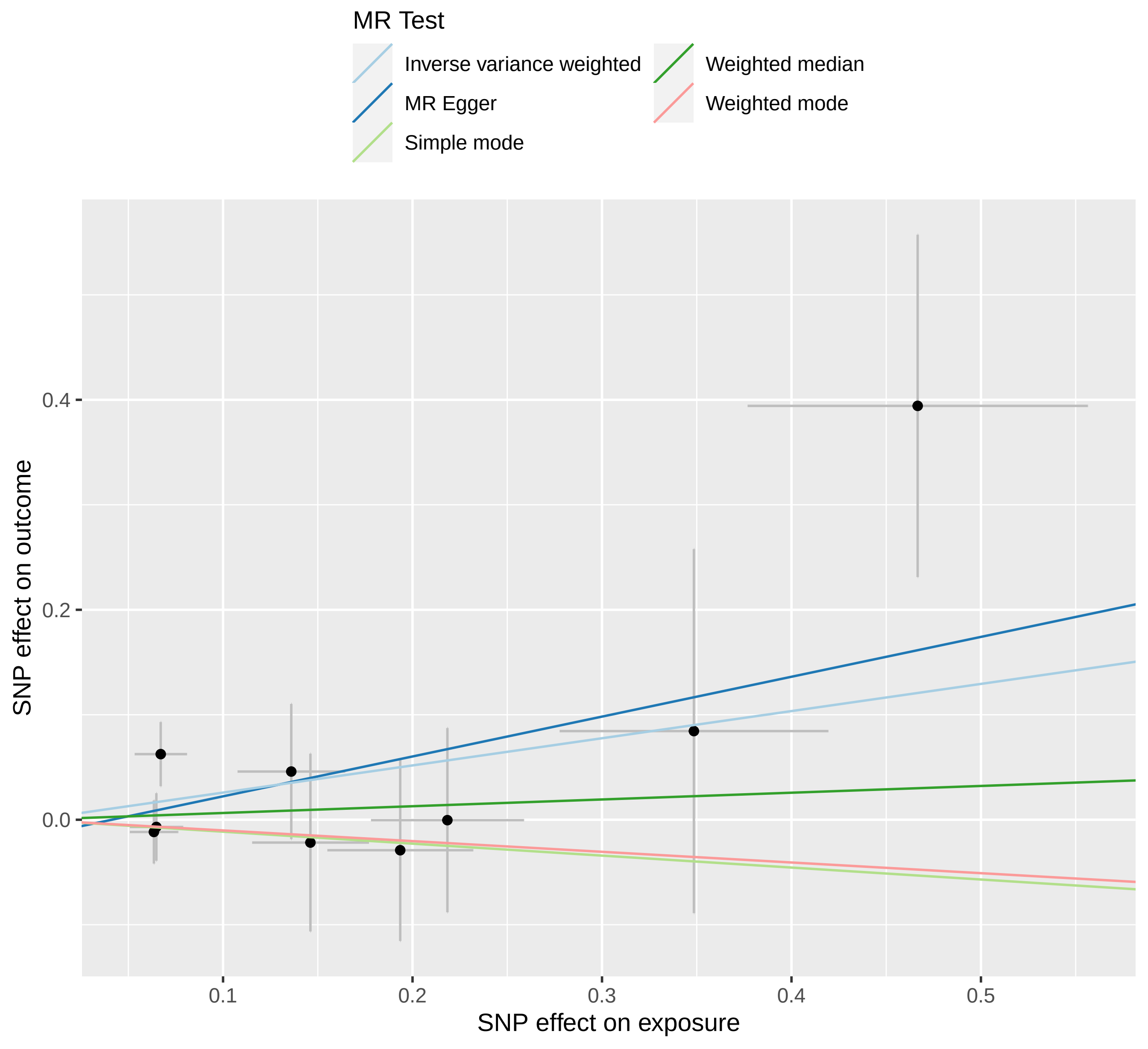
MR Test
Inverse variance weighted
MR Egger
Simple mode
Weighted median
Weighted mode
SNP effect on outcome
0.4
0.2
0.0
0.1
0.2
0.3
0.4
0.5
SNP effect on exposure
